# Patterns of enhancement in paretic shoulder kinematics after stroke with musical cueing

**DOI:** 10.1038/s41598-020-75143-0

**Published:** 2020-10-22

**Authors:** Shinil Kang, Joon-Ho Shin, In Young Kim, Jongshill Lee, Ji-Yeoung Lee, Eunju Jeong

**Affiliations:** 1 Department of Biomedical Engineering, Hanyang University, Seoul, South Korea; 2 National Rehabilitation Centre, Seoul, South Korea; 3 Department of Medical Science, Graduate School, Soon Chun Hyang University, Asan, South Korea; 4 Department of Music and Science for Clinical Practice, Hanyang University, Seoul, South Korea; 5 College of Interdisciplinary Industrial Studies, Hanyang University, Seoul, South Korea

**Keywords:** Rehabilitation, Rehabilitation

## Abstract

Musical cueing has been widely utilised in post-stroke motor rehabilitation; however, the kinematic evidence on the effects of musical cueing is sparse. Further, the element-specific effects of musical cueing on upper-limb movements have rarely been investigated. This study aimed to kinematically quantify the effects of no auditory, rhythmic auditory, and melodic auditory cueing on shoulder abduction, holding, and adduction in patients who had experienced hemiparetic stroke. Kinematic data were obtained using inertial measurement units embedded in wearable bands. During the holding phase, melodic auditory cueing significantly increased the minimum Euler angle and decreased the range of motion compared with the other types of cueing. Further, the root mean square error in the angle measurements was significantly smaller and the duration of movement execution was significantly shorter during the holding phase when melodic auditory cueing was provided than when the other types of cueing were used. These findings indicated the important role of melodic auditory cueing for enhancing movement positioning, variability, and endurance. This study provides the first kinematic evidence on the effects of melodic auditory cueing on kinematic enhancement, thus suggesting the potential use of pitch-related elements in psychomotor rehabilitation.

## Introduction

Stroke is a leading cause of long-term functional disability^1^, resulting in increased dependency^2^ and social isolation^3,4^ as well as decreased quality of life of patients^5,6^. Approximately 80% of stroke survivors have upper-limb dysfunction, varying from gross to complex and fine motor movements^7^. Post-stroke upper-limb rehabilitation remains challenging^8–11^, and the diverse approaches and methodologies have yielded controversial results^11,12^. Recently, physicians and researchers have explored the potential of using diverse types of sensory cueing as a complement to conventional, vision-oriented approaches for motor rehabilitation^13–15^. Auditory stimulation has especially shown beneficial effects in enhancing movement execution^16–20^. Such stimuli may be perceived from all directions and reach across distances, making them perceptible even when the patient is not consciously or attentively listening^21^. Further, they immediately increase the excitability and readiness of motor execution, entrain the periodicity of movement patterns, and allow individuals to anticipate and prepare for the forthcoming movement^22,23^.

Auditory-motor enhancement is believed to have multifaceted mechanisms. At the perceptual level, auditory stimuli reach the brain faster than visual and tactile stimuli^24–26^. The auditory system has a high temporal resolution^27,28^ and easily synchronises with the temporal periodicity of movements^29,30^. At the neuronal level, auditory cueing modulates neuromagnetic β oscillations^31,32^, recruits movement-specific motor neurons^33^, and increases the variability in musculoskeletal activation patterns^34^. Most important, neural substrates involved in auditory processing closely communicate with those modulating motor timing, sequencing, and execution^35–37^. This is evidenced by the presence of a cortico-subcortical network that involves the putamen, supplementary motor area, premotor cortex, and auditory cortex^38–41^. Such auditory-motor connectivity is well represented and adopted in clinical studies. For example, rhythmic auditory stimulation (RAS), one of the techniques used in neurologic music therapy, leads to enhanced lower- and upper-extremity movements in patients with neurological impairments^42–44^. Specific to the upper limbs, movement timing^45^ and movement trajectory smoothness^46^ and velocity^45,47^ were greatly improved when RAS training was used in previous studies.

However, research on pitch-related elements has been relatively sparse. Perception of pitch and melody is part of the intrinsic nature of humans, is based on tonotopic representation in the auditory cortex^48–50^, and is associated with space perception^51,52^. Thus, pitch and melody can provide a cognitive representation of movement in terms of spatial location and direction^53^. For example, ascending and descending melodic contours cue upward and downward movements. The effects of pitch-related elements have also been demonstrated in clinical studies. Patterned sensory enhancement utilises rhythmic, melodic, and harmonic elements to provide temporal, spatial, and dynamic information about the movement^54,55^ and has been applied to upper-limb rehabilitation^55–61^. However, the results have been inconsistent, which might be due to the non-specific use of musical elements. Moreover, to the best of our knowledge, element-specific effects have not been examined using a controlled experimental design to date.

Additionally, most previous studies employed conventional observations, rating scales, or questionnaires for evaluation^56–60^, which are inherently subjective and vary depending on the evaluator(s)^62^. These methodologies hardly allow the measurement of kinematic changes throughout upper-limb movements during auditory cueing; thus, more sophisticated methodologies are needed. In the present study, we employed inertial measurement units (IMUs) to quantify the kinematics of repetitive shoulder abduction, holding, and adduction movements in hemiparetic stroke patients. The purpose of the present study was to examine the effects of (1) no auditory cueing (NAC), (2) rhythmic auditory cueing (RAC), and (3) melodic auditory cueing (MAC) on the kinematic parameters of shoulder movement, including range of motion (ROM), minimum Euler angle (MIN), maximum Euler angle (MAX), duration, and root mean square error (RMSE).

## Results

**Figure 1:**
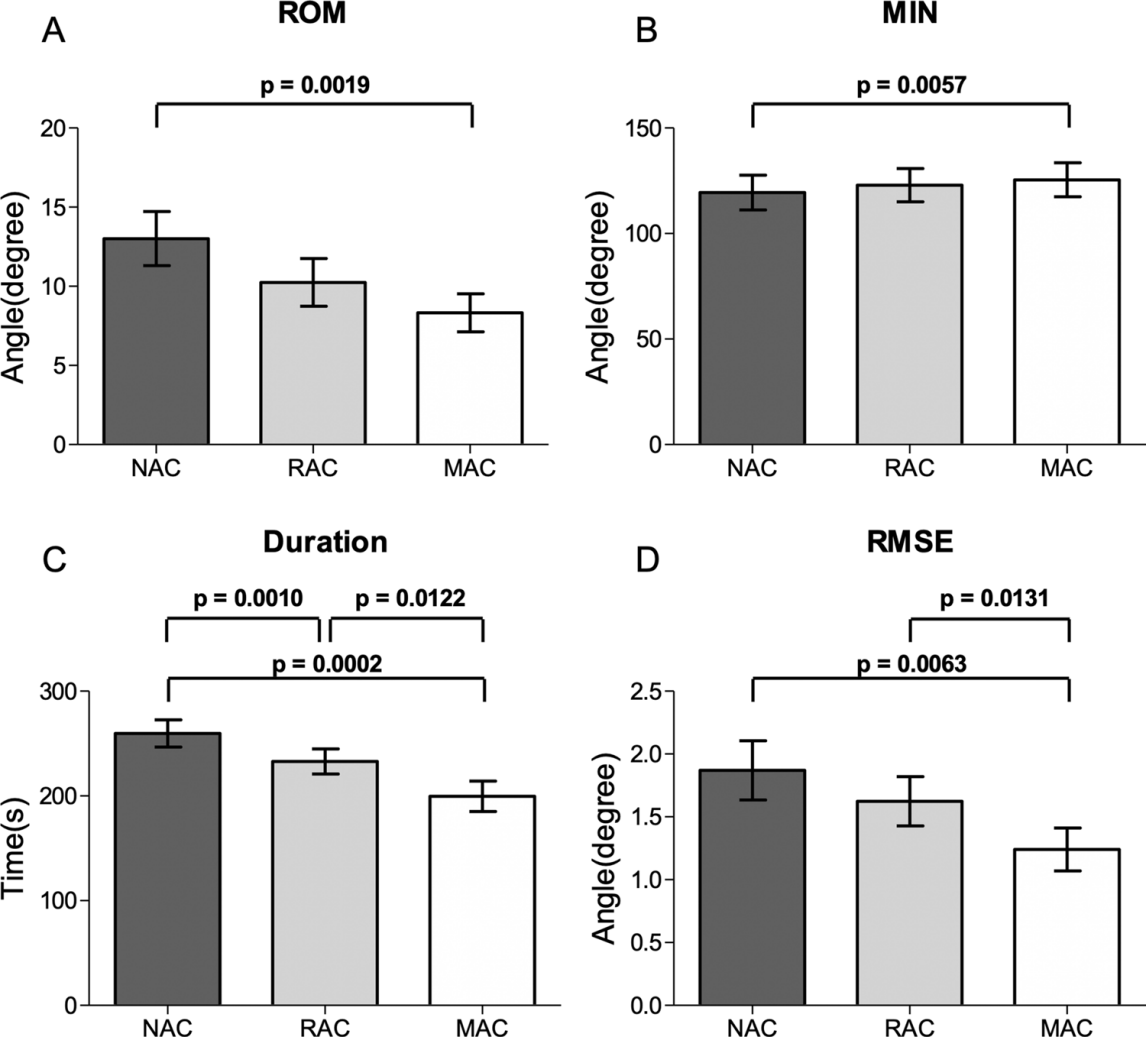
Significant differences of kinematic parameters of movement. The graphs show means with standard errors. (**A**) ROM during the holding phase. (**B**) MIN during the holding phase. (**C**) Duration during the holding phase. (**D**) RMSE during the holding phase. *P* values are provided for significant differences (*p* < 0.0167).

Table 1 shows the descriptive findings. During the holding phase, there existed a significant main effect of the cue on the ROM [*F*_(2,30)_ = 8.801, *p* < 0.01] and MIN [*F*_(2,30)_ = 9.087, *p* < 0.01] (Fig. 1). A pairwise post-hoc comparison with Bonferroni correction revealed that the ROM was greater in NAC than in MAC (*p* < 0.0167), whereas the MIN was higher in MAC than in NAC (*p* < 0.0167). A higher MIN indicated that MAC can assist in the maintenance of shoulder holding at a higher position. The smaller ROM observed in MAC than in NAC and RAC indicated that MAC helped maintain a less variable shoulder angle with less Euler angle drift.

**Table 1:** Descriptive statistics of kinematic parameters.

Kinematic parameter	Abduction			Holding			Adduction		
	NAC	RAC	MAC	NAC	RAC	MAC	NAC	RAC	MAC
**ROM**									
M	127.27	128.61	128.33	13.01	10.25	8.32	119.88	121.55	123.57
SD	53.58	52.33	53.35	11.84	10.42	8.32	56.91	54.82	55.91
**MIN**									
M	4.63	5.15	5.33	119.43	122.95	125.48	4.65	5.01	5.00
SD	10.53	12.41	11.12	57.00	54.63	55.98	9.89	12.52	10.85
**MAX**									
M	131.91	133.79	133.68	135.63	135.78	135.50	124.60	126.67	128.60
SD	54.48	52.29	53.79	54.74	52.10	54.22	57.87	54.38	56.14
**RMSE**									
M	11.91	12.97	12.78	1.87	1.62	1.24	13.37	13.08	13.69
SD	8.63	8.67	8.21	1.64	1.36	1.18	10.96	9.60	9.91
**Duration**									
M	295.44	300.36	312.59	259.60	232.90	199.63	330.24	351.84	373.36
SD	66.57	64.22	64.38	90.02	82.63	55.01	73.95	90.27	93.52

*ROM* range of motion, *MIN* minimum Euler angle, *MAX* maximum Euler angle, *RMSE* root mean square error, *NAC* no auditory cueing, *RAC* rhythmic auditory cueing, *MAC* melodic auditory cueing, *M* mean, *SD* standard deviation.

Further, there existed a significant main effect of cue on the duration [*F*_(2,30)_ = 22.028, *p* < 0.001]. A pairwise comparison showed that during the holding phase, the duration of shoulder movement was significantly longer in NAC than in RAC (*p* < 0.0167), in NAC than in MAC (*p* < 0.0167), and in RAC than in MAC (*p* < 0.0167). Interestingly, there also existed a significant main effect of cue on the duration [*F*_(2,30)_ = 6.724, *p* < 0.01] in the adduction phase. Although the pairwise comparison yielded insignificant results for all pairs (*p* > 0.0167), the duration associated with MAC was the longest, followed by that associated with RAC and then NAC.

We also performed a movement variability (MV) analysis. General approaches to MV quantification in upper-limb movements are typically based on the distribution of angle, acceleration, and velocity^63^. During the holding phase, there was a significant main effect of cueing on the RMSE [*F*_(2,30)_ = 8.109, *p* < 0.01]. A pairwise post-hoc comparison revealed significantly smaller RMSE values in MAC than in NAC (*p* < 0.0167) and RAC (*p* < 0.0167) (Fig. 1).

**Figure 2:**
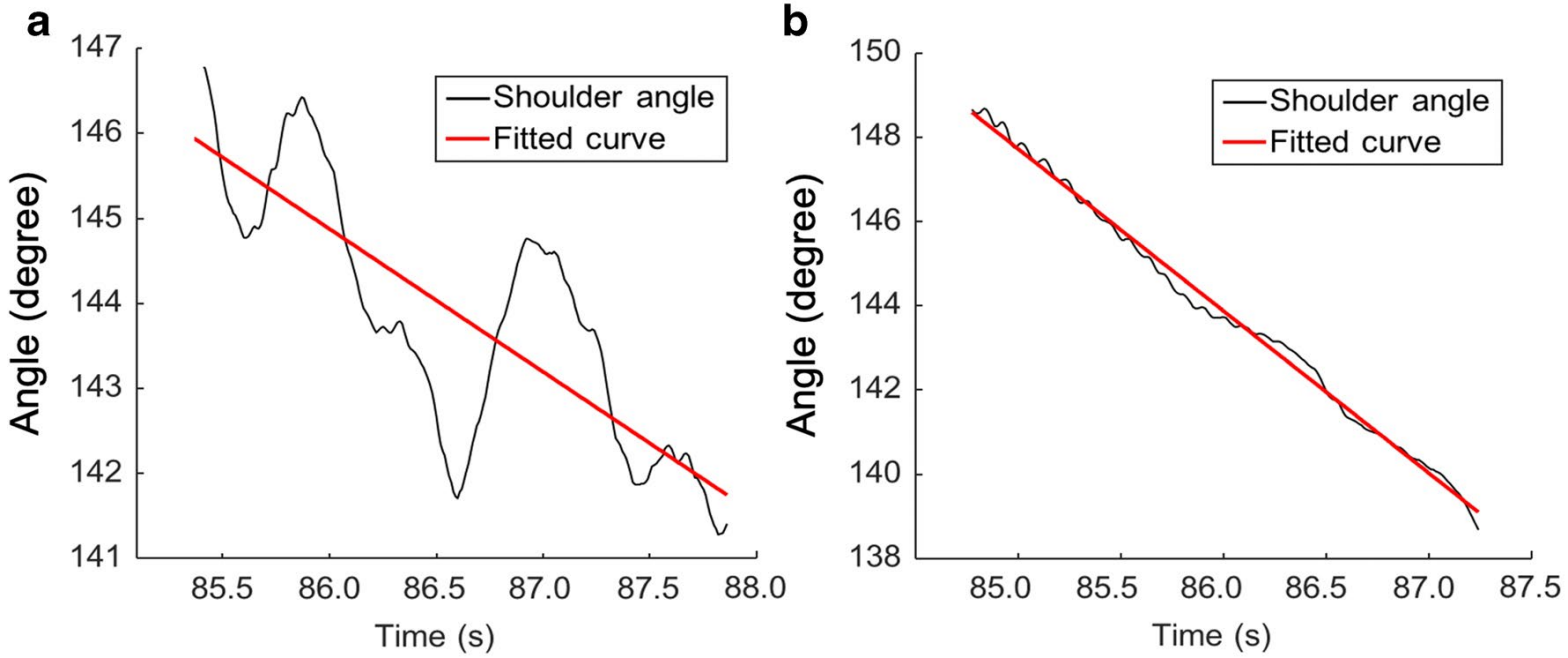
Comparison of Euler angle data of shoulder movement. (**A**) Data obtained from severely affected patients’ shoulders. (**B**) Data obtained from moderately affected patients’ shoulders.

We calculated RMSE as a MV indicator because Euler angle values are sensitive for the measurement of movement acceleration and deceleration. Figure 2 shows the Euler angle variability between the affected and intact sides (black line) during the holding phase, as compared with the fitted curve (red line). The Euler angle of the affected side showed a sudden decrease after the arm approached the highest position and before the start of the adduction phase. This finding was similar to observations made in previous studies that investigated the stroke-specific kinematic features of shoulder movements^63–66^.

**Figure 3:**
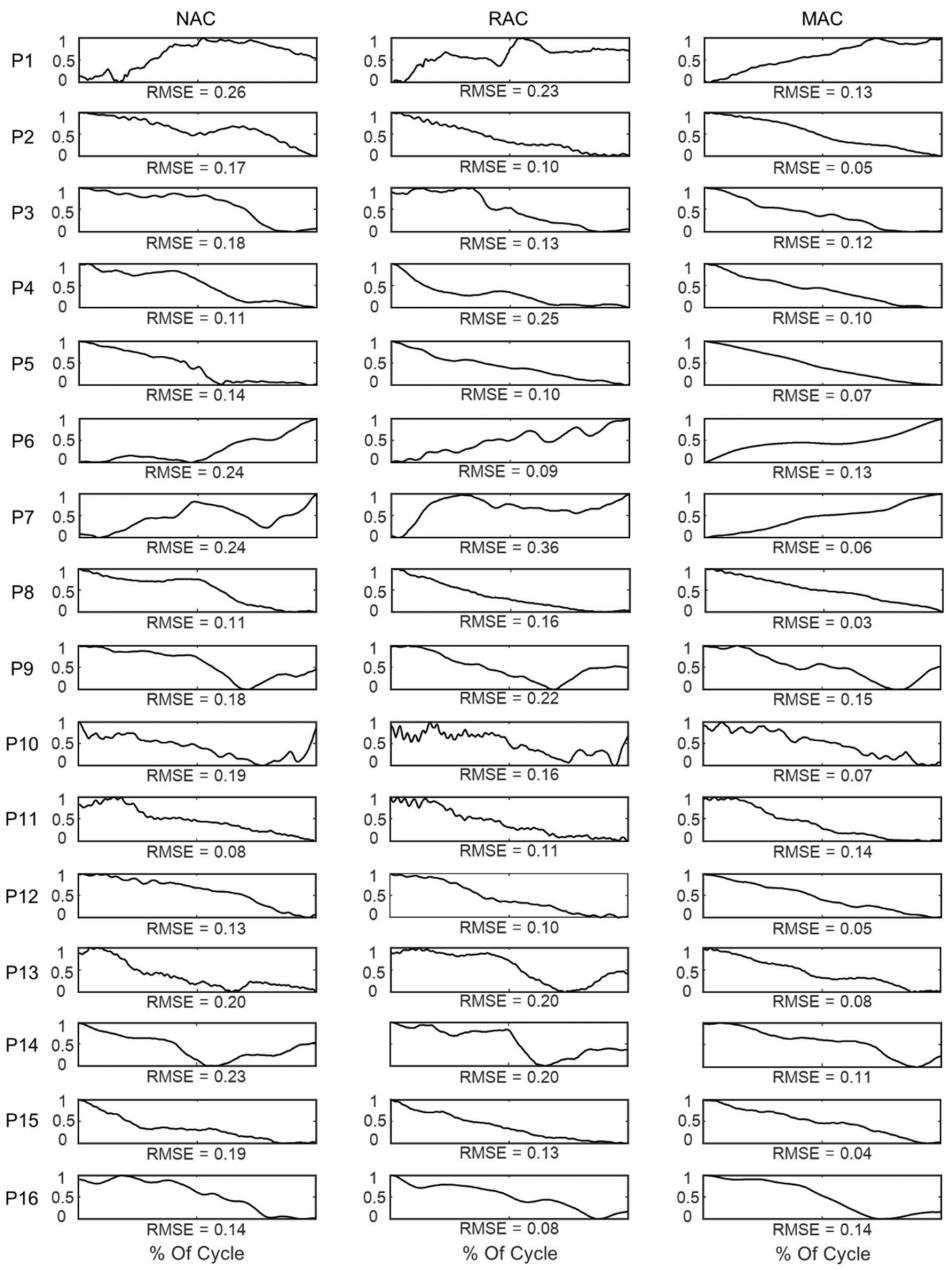
Movement variability profile using Euler angle data. Representative RMSE profiles of the magnitudes of the Euler angle of 16 patients during the shoulder movement holding phase are shown. The x-axis displays 100% of the movement cycle: 0% and 100% are the shoulder movement holding times. The y-axis displays the normalised Euler angle values. Normalised Euler angle values were used to enhance the comparison among patients. The left column shows the NAC condition; the middle column shows the RAC condition; and the right column shows the MAC condition.

Further, we scrutinised the Euler angle profiles during the holding phase. Figure 3 shows representative Euler angle magnitude profiles. A visual inspection revealed that, in all patients except P2 and P11, less Euler angle profile variability was observed in MAC than in NAC. Moreover, in most patients except P2, P9, P10, P11, and P16, less Euler angle profile variability was observed in MAC than in RAC.

## Discussion

In the present study, we used IMUs to measure the complex kinematic characteristics of shoulder abduction, holding, and adduction movements occurring in conjunction with NAC, RAC, and MAC. The findings revealed that MAC significantly increased the MIN and decreased the ROM and RMSE during the holding phase. MAC also decreased the duration of the holding phase. Taken together, these findings indicated that MAC enhances movement positioning, decreases movement variability, and increases movement anticipation and preparation.

First, the results during the holding phase showed that the MIN was significantly higher in MAC than in NAC and that the ROM was significantly greater in NAC than in MAC. These findings collectively indicated that the MIN of the shoulder was maintained at the highest position with less movement deviation in MAC. The enhancement observed in MAC occurred because the participants were provided with information about movement directions until they reached their maximum shoulder angle. These results are similar to those of previous studies that examined the effects of music cognition on movement, demonstrating that pitch contours can increase the cognitive awareness of spatial information during motion^51^ and actually enhance limb positioning during the execution of vertical movements^67^.

Second, the RMSE was significantly smaller in MAC than in NAC and RAC during the holding phase. These findings indicated that the observed Euler angle data, which can sensitively detect movement acceleration and deceleration, were best fitted to the predicted linear regression model in MAC; thus, MAC yielded the smallest RMSE. Note that during the holding phase, MAC involved an isochronous rhythm combined with a 740-Hz stationary contour, which is higher than the tone used in RAC (440 Hz, see Fig. 1). Thus, the closer RMSE values of the observed data to the predicted model in MAC was possibly due to the effect of high-pitched sequences embedded in the middle of a melodic contour on the cognitive representation of movement in terms of spatial location^53^, which allowed the participants to sustain their shoulders with less movement variability. In line with the current findings, a previous study reported that a melody cue was best for unconstrained point-to-point hand movements, prompting less variable hand movements from the initial to the final position within a given time period^68^. Moreover, previous studies have demonstrated that auditory cueing significantly decreases movement variability^45,46,69–72^. For example, Thaut et al. reported that the kinematic data obtained in auditory cueing fit the predicted model significantly better than those from a no auditory cueing condition. This result was possibly due to the entrainment of movement execution patterns with the patterns embedded in the rhythm^46^.

Third, the duration was significantly shorter in MAC than in NAC, in RAC than in NAC, and in MAC than in RAC. RAC consists of an isochronous rhythm, whereas MAC consists of pitch contours presented with an isochronous rhythm. In the current study, this rhythm pattern seemed to play a role in movement anticipation and preparation in both RAC and MAC. In a review study, Avanzino et al.^73^ reported that rhythm provides a temporal structure that enhances motor timing, motor sequencing, and movement control. Moreover, isochronous rhythm was reported to induce a temporal locking or entrainment process between motor movements and the external auditory rhythm^22^.

In addition, the shorter duration of RAC and MAC can be interpreted as a cognitive influence of musical cueing on anticipation and preparation for movement. Schaffert et al.^74^ proposed that auditory rhythm can prime the motor system and facilitate further anticipation of and preparation for cyclic movements^75^. Such influences, when observed in healthy and clinical (e.g. post-stroke) populations, manifest through the activation or establishment of alternative pathways^76–78^. The available findings implied that cognitive involvement in auditory processing (i.e. anticipation) plays a role in motor preparation and execution^79,80^. Importantly, as the pitch contours embedded in RAC are more salient to providing the structure of movement, patients are more likely to anticipate and prepare for the forthcoming movement. In general, music involves a high-order temporal organisation of sound events, of which allows individuals to make predictions about future events and raises expectations^37^. Previous studies that employed melodic feedback in post-stroke arm rehabilitation also reported improved smoothness of movement because of such anticipation^81,82^.

Collectively, MAC led to an effective, stable maintenance of the shoulder in the desired position during the holding phase. The closer fit observed in MAC indicated a smaller movement variability possibly due to the effect of pitch in addition to the effect of rhythm. The shorter movement time in MAC indicates that rhythm components play a role in movement anticipation and preparation and are more prominent when presented with melodic components. Compared with MAC, RAC showed significant enhancement only in movement time.

Our study had some limitations. We performed parameter extraction for the Euler angles of the sagittal axis because this axis provides more relevant information about our target movement. Obtaining similar data from two other axes and involving another body part (wrist, hand, neck, etc.) may provide a more comprehensive index for the movement features of the affected shoulder (e.g. post-stroke compensatory strategies). However, this study was a preliminary attempt to quantify the kinematic parameters of repetitive paretic shoulder abduction, holding, and adduction in post-stroke patients. As the effects of musical cueing on the kinematic changes of upper-limb movements have not yet been specified and the scope of their application has been limited, the present study provides important information about the specific roles of the elements as sensory cues and promotes a more appropriate use of the elements for upper-limb rehabilitation. Future studies are needed to find a more comprehensive index of the relationship between musical cueing and functional upper-limb movements and to develop a more efficient and targeted upper-limb patterned sensory enhancement protocol. Lastly, combining musical cueing with visually oriented virtual reality rehabilitation may yield better psychomotor rehabilitation.

In conclusion, auditory cueing led to enhanced movements, across all parameters, when compared with movements performed without auditory cueing in this study. In particular, MAC effectively increased the MIN but decreased the RMSE and duration in the holding phase. These findings seem to be associated with increased movement positioning, decreased movement variability, and enhanced movement anticipation and preparation. Such findings suggest the potential of pitch-related components for directing specific movement parameters and the potential use of musical cueing in psychomotor rehabilitation. Given that muscle endurance is necessary to allow smooth control of movements, which is typically impaired in stroke patients (as evidenced by their daily functional movements)^83–85^, the current findings can be used for the conclusive measurement of motor function recovery and for rehabilitation training^86^.

## Methods

### Participants

This study was approved by the Institutional Review Board at the National Rehabilitation Centre and performed in accordance with relevant guidelines and regulations. All participants provided written informed consent, in accordance with the Declaration of Helsinki. Informed consent to publish identifying images was also obtained from participants. Eighteen patients with hemiparetic stroke (Male = 10, Female = 8) volunteered to participate in the study and were recruited from the rehabilitation centre. The patients were eligible if they had upper-limb hemiparesis secondary to first-ever ischaemic or haemorrhagic stroke, Brunnstrom Stages of Motor Recovery scores > 2 for the affected proximal upper limb, the cognitive ability to understand and follow instructions, and the capability and willingness to participate in the experiment. Patients who had more than 3 months of regular involvement in musical activities and/or professional training and those with sensory impairments were excluded from the study. Two of the 18 patients were excluded because of unreliable signal-to-noise ratios. The average age of the patients was 49.78 (SD = 15.55) years, and the average time since stroke was 15.22 (SD = 12.82) months. The patients had an average of 12.28 (SD = 2.80) years of education, an average Brunnstrom Stage of Motor Recovery score of 4.67 (SD = 0.94), and an average shoulder abduction ROM of 138.33° (SD = 29.06).

### Movement task and auditory stimuli

To examine the immediate effects of auditory cueing on paretic shoulder movements, we selected shoulder abduction, holding, and adduction movements as previous studies had reported the stroke-specific kinematic features of shoulder movements^87–90^. A visual avatar, from a third-person perspective, was provided to guide the movement. The avatar was programmed to raise its arms from the side to 180° (abduction phase, 3 s), maintain its arms at that position (holding phase, 3 s), and then slowly lower its arms back to the side (adduction phase, 3 s). The participants were instructed to perform the movements in conjunction with the avatar seen on the computer screen. The avatar was generated using a model of Rehab Master (Rehab Master, Seoul, Korea) and programmed using Unity (Unity Technologies, Copenhagen, Denmark).

Two types of auditory stimuli were used as auditory cues and presented via two stereo speakers. RAC consisted of a series of 50-ms tones with an inter-stimulus interval (ISI) of 200 ms (i.e. a beep sound on digital metronome at an audio frequency of 440 Hz). MAC consisted of a series of tones presented using the same isochronous time pattern as the RAC but at different frequencies. In the present study, RAC and MAC have definite pitches and the same isochronous time pattern, so they might also be labelled *monotonic rhythm* and *melodic rhythm*, respectively. However, we used the terms *rhythmic* and *melodic* in accordance with the terminologies used in the existing literature. RAC or RAS is a frequently used psychomotor rehabilitation technique. The rhythmic stimuli employed in these studies are mainly analogue or digital metronome beats^69,76,91^ comprising tones presented at the same pitch and with an isochronous time pattern. In addition, considering the general definition of *melody* as a combination of pitch and rhythm, it might be better to use the term MAC.

**Figure 4:**
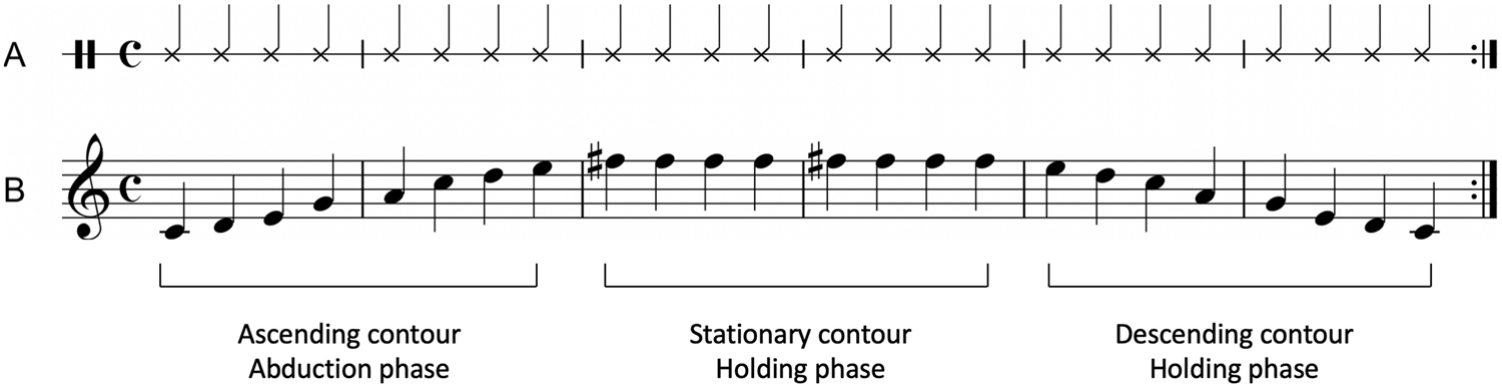
An example of auditory cueing. (**A**) Rhythmic auditory cueing. (**B**) Melodic auditory cueing.

MAC consisted of ascending, stationary, and descending contours generated to musically demonstrate the angle of shoulder abduction, holding, and adduction, respectively. In typical participants, shoulder abduction involves three different types of muscle—the supraspinatus, deltoid, and trapezius and serratus anterior. The supraspinatus initiates from 0° to 15°, the deltoid functions from 15° to 90°, and the trapezius and serratus anterior are activated from 90° to 180°^92^. In addition, the glenohumeral joint contributes 90°–120° of shoulder abduction^93,94^. The range of shoulder movement (0°–180°) was divided into ten equal degrees, and a 12-tone scale (C4 to F#5) was assigned to each degree. As 0°, 15°, 90°, 120°, and 180° are important to indicate the muscle involvement in shoulder abduction, we selected the pitches that correspond to the degrees (C4, D4, A4, C5, and F5#). As 15° corresponds to the microtone between C4# and D4, we selected D. Additional tones were used to musically describe the spatial position and trajectory of shoulder movement, yielding an eight-tone ascending contour. The first four tones of the ascending contour (C4, D4, E4, and G4) were assigned to shoulder abduction from 0° to 89°, and the second four tones (A4, C5, D5, and E5) were assigned to shoulder abduction from 90° to 179°. The next eight tones of the stationary contour reflect the shoulder holding movement. As maximum effort is needed to hold the shoulder at the highest position (typically around 170°–180°), F5# was used to reflect a such effort. Figure 4 shows an example of RAC and MAC. The auditory stimuli were generated using a musical instrument digital interface synthesizer connected to a Logic Pro X (Apple, Cupertino, CA, USA) and programmed in accordance with the avatar movements using Unity (Unity Technologies, Copenhagen, Denmark).

### IMUs and equipment setup

**Figure 5:**
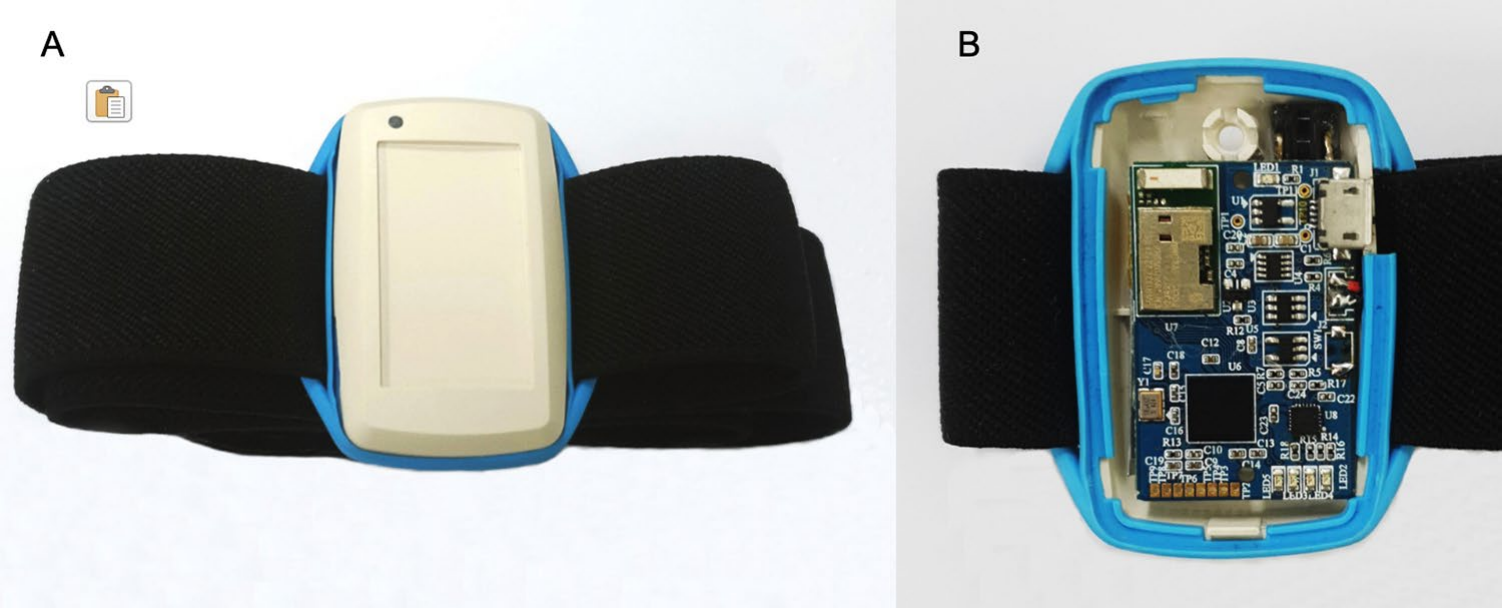
In-house-built H-IMU. (**A**) Cover closed. (**B**) Cover opened.

**Figure 6:**
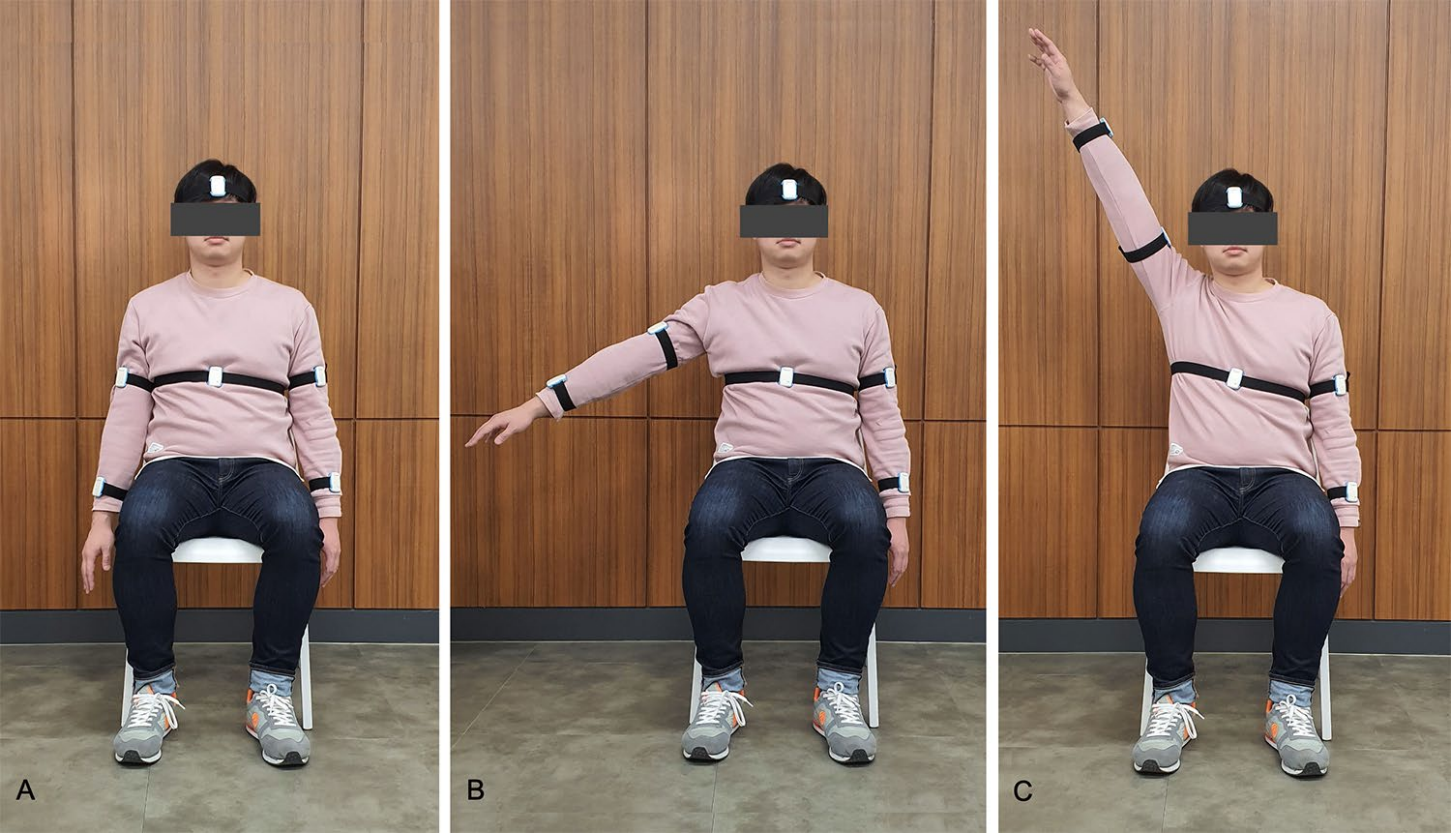
An individual demonstrating shoulder abduction with the H-IMUs attached. (**A**) Arm at the side. (**B**) Raising one arm halfway. (**C**) Reaching the arm towards the head.

To measure and analyse the effects of cueing on the participants’ shoulder movements, we used spatiotemporal kinematic parameters. These parameters were provided by Hanyang IMU (H-IMU), which consists of an integrated 9-axis motion tracking sensor (MPU9250; InvenSense, San Jose, CA, USA), a microcontroller (MSP430F5338; Texas Instruments, Dallas, TX, USA), a Bluetooth module (PAN1321i; Panasonic, Osaka, Japan), and a lithium-ion battery (240 mAh, 3.7 V; DTP, Shenzhen, China). The device allows long-term data measurements (Fig. 5). A total of 6 H-IMUs were attached to the head, torso, arms, and forearms of each patient using Velcro straps and were positioned on the dorsal side of each arm and on the anterior portion of the head and torso. Signals associated with body movements were generated at < 20 Hz, such that a sampling frequency of 100 Hz was sufficient to detect all movements. Thus, all H-IMU signals were measured using a sampling rate of 100 Hz. The calculated Euler angles for each of the 6 H-IMUs were transmitted to a personal computer using Bluetooth communication. Figure 6 shows the H-IMUs and how they were attached to a participant.

In each experiment, the participants underwent a practice period (P1) and an experimental period (P2). P1 comprised three shoulder abduction-holding-adduction trials, in which the three types of cueing were presented. The P1 period allowed the participants to become familiar with the movements and the different types of cueing. P2 comprised three blocks of three cueing conditions. Five trials, each consisting of abduction, holding, and adduction phases, were blocked with one of the three cueing conditions and randomly presented to the participant while avoiding the consecutive presentation of the same cueing condition. The blocks requiring the movement of the affected and unaffected shoulders were presented in an alternating manner.

### Euler angle calculation

Micro-electromechanical system inertial sensors have a direct-current bias and gain error because of the direct-current noise caused by powering of the electric circuit and environmental noise. To minimise the error associated with these noises, we calibrated the sensors using an in-house-manufactured calibration jig with 9 degrees of freedom (DoFs)^95^. The calibrated data, c, can be obtained by multiplying the uncalibrated data, u, including the original error by the compensated gain value, K, through calibration and subtracting the offset error, b. These variables are defined in Eqs. (1–3), respectively. Finally, the calculated calibration equation is shown in Eq. (4)

1 $$ u = \left[ {\begin{array}{*{20}l} {u_{x} } \hfill & {u_{y} } \hfill & {u_{z} } \hfill \\ \end{array} } \right]^{T} $$

2 $$ {\text{K}} = { }\left[ {\begin{array}{*{20}c} {k_{x} } & 0 & 0 \\ 0 & {k_{y} } & 0 \\ 0 & 0 & {k_{z} } \\ \end{array} } \right] $$

3 $$ {\text{b}} = \left[ {\begin{array}{*{20}l} {b_{x} } \hfill & {b_{y} } \hfill & {b_{z} } \hfill \\ \end{array} } \right]^{T} $$

4 $$ {\text{c}} = {\text{Ku}} - {\text{b}} $$

**Figure 7:**
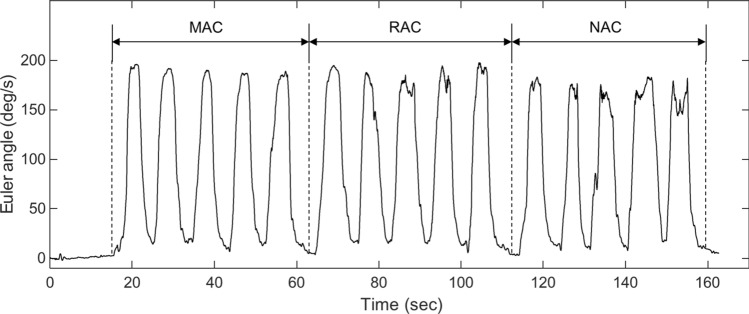
Kinematic recording of hemiparetic shoulder abduction, holding, and adduction movements across three types of cueing.

Thereafter, the Euler angles were calculated using a gradient descent algorithm to provide the quaternions based on tri-axis gyroscope, accelerometer, and magnetometer measurements, as shown in Eq. (5). Euler angles adopt the ‘ZYX’ rotation sequence to convert quaternion frame rotations in radians^96,97^. Figure 7 shows an example of the calculated Euler angles associated with the three types of cueing

5 $$ \begin{aligned} & \emptyset = {\text{atan}}2\left( {2{\text{q}}_{3} {\text{q}}_{4} - {\text{q}}_{1} {\text{q}}_{2} ,\;2{\text{q}}_{1}^{2} + 2{\text{q}}_{4}^{2} - 1} \right) \\ & \theta = - {\sin}^{ - 1} \left( {2{\text{q}}_{2} {\text{q}}_{4} + 2{\text{q}}_{1} {\text{q}}_{3} } \right) \\ & \varphi = {\text{atan}}2\left( {2{\text{q}}_{2} {\text{q}}_{3} - 2{\text{q}}_{1} {\text{q}}_{4} ,\;2{\text{q}}_{1}^{2} + 2{\text{q}}_{2}^{2} - 1} \right) \\ \end{aligned} $$

where ${\mathrm{q}}_{\mathrm{x}} (\mathrm{x}=1, 2, 3, 4)$ is the estimated orientation of the sensor frame relative to the earth frame.

### Movement analysis

**Figure 8:**
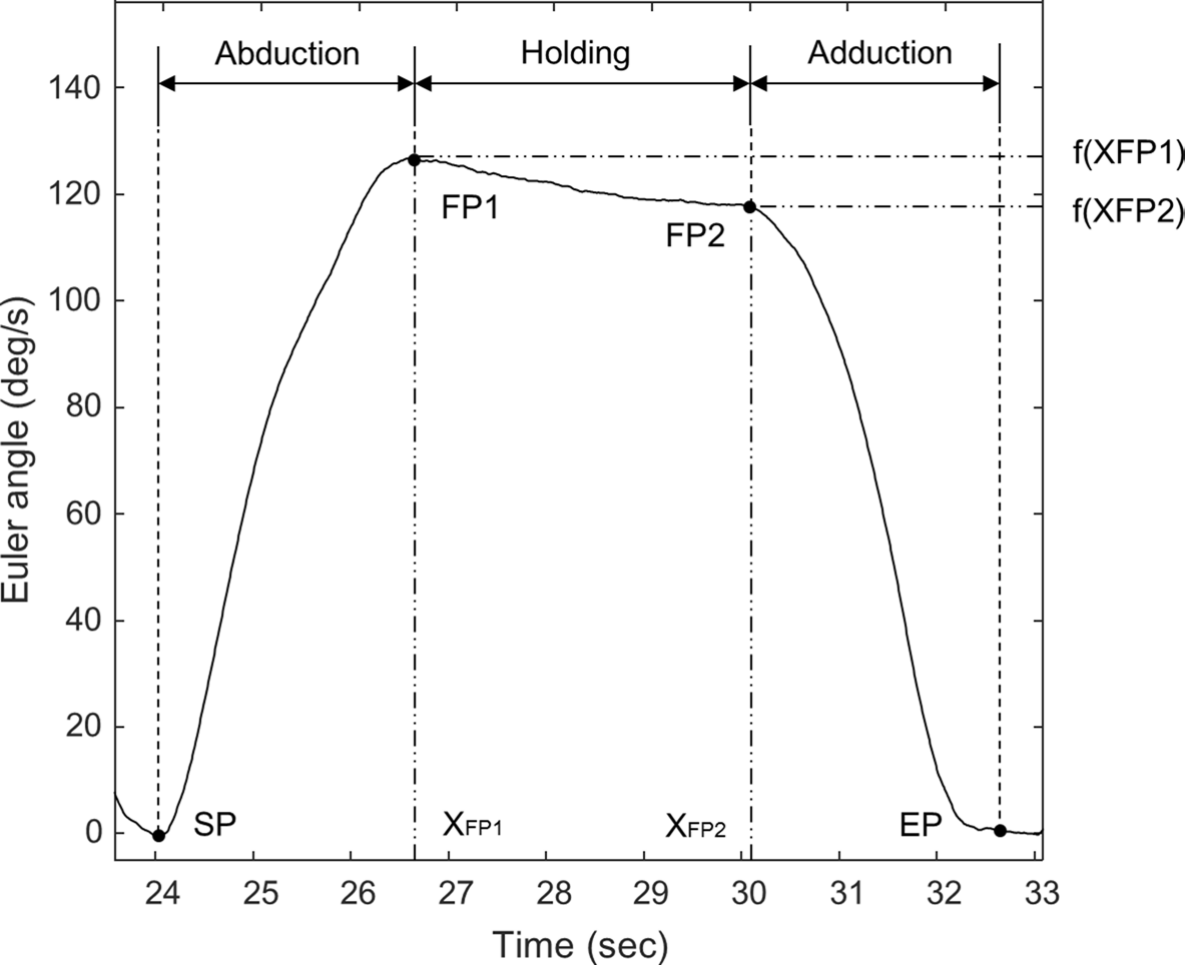
Sample raw Euler angle data obtained from one trial of shoulder abduction, holding, and adduction. *SP* start point, *EP* end point, *FP* fiducial point.

We extracted the kinematic parameters of movement during the three phases (abduction, holding, and adduction) of shoulder movement. Parameter extraction was performed on the Euler angles obtained from the frontal plane around the sagittal axis, which is the axis that provides more relevant information about the abduction, holding, and adduction movements of the shoulder. Figure 8 shows the points that were included in the movement analysis. The start point (SP) and end point (EP) indicate the initiation of the abduction phase and the cessation of the adduction phase, respectively, based on the minimum Euler angle values. Fiducial points (FP1, FP2) were defined to correspond with the initiation and cessation of the holding phase; FP1 represents the maximum point on the Euler angle graph for one cycle.

We selected FP2 values that satisfy the equations below, in which *f*(*x*) represents the angle, *x* represents the time point, and i represents the peak points between FP1 and EP. FP2 was not easy to define because the signal-to-noise ratios were extreme in all participants. Based on our preliminary experimentation and simulations, we set a threshold (α) for the between-peak slope (− 0.03) and applied this to all data sets. Small peak points, with peak values below − 0.03, were not considered to be reliable FP2 values (6, 7)

6 $$ {\text{P}} = \left\{ {x_{i} \left| {\frac{{f(x_{FP1} ) - f(x_{i} )}}{{x_{FP1} - x_{i} }} < {\upalpha },} \right.\quad {\text{FP}}1 \le {\text{i}} \le {\text{EP}}} \right\} $$

7 $$ x_{FP2} = \max \left( P \right),\;y_{FP2} = f(x_{FP2} ),\;FP2 = (x_{FP2} , y_{FP2} ) $$

Based on previous studies in clinical populations that revealed the meaning of Euler angle values relative to motor movements^98–100^, we calculated the kinematic parameters using Euler angles (Table 2).

**Table 2:** Spatiotemporal features.

Kinematic parameter	Formula	Description
ROM	$\mathrm{max}\left({\varnothing }_{phase}\right)-\mathrm{min}({\varnothing }_{phase})$	
MIN	$\mathrm{min}({\varnothing }_{phase})$	
MAX	$\mathrm{max}({\varnothing }_{phase})$	
RMSE	$\sqrt{\frac{1}{n}\sum_{i=FP1}^{FP2}{({\varnothing }_{i}-{\widehat{\varnothing }}_{i})}^{2}}$	Calculated using a linear regression model
Duration	${t}_{end phase}- {t}_{start phase}$	Time spent to complete one phase of each trial

${\mathrm{\varnothing }}_{\mathrm{phase}}$ Euler angle values obtained during one phase of each trial, *n* number of samples for the phase, $\widehat{\varnothing }$ linear regression value, *t* time point, *MIN* minimum Euler angle of the phase, *MAX* maximum Euler angle of the phase, *RMSE* root mean square error.

#### ROM

Shoulder movement is characterised by many DoFs and a wide ROM at the joints^101^. The ROM is important for assessing joint range, control, strength, and willingness to perform a movement^102^. The clinical implication of the kinematic ROM has recently been expanded to the determination of diagnoses and the success of therapeutic interventions. Also, previous studies have reported the validity and reliability of IMUs for measuring parameters of shoulder joint motion, such as the ROM^103–105^. In the present study, the ROM refers to the difference between the MIN and MAX, using the anatomical DoFs for human limbs^64,106^, and was calculated for each of the three movement phases. In the abduction and adduction phases, the ROM reflects the largest Euler angle, as arm raising was initiated from approximately 0°. During the holding phase, the ROM reflects the variance among the Euler angles.

#### Movement variability (MV)

General approaches to MV quantification in upper-limb movements are typically based on the distribution of characteristic values, such as angle, acceleration, and velocity^63^. Movement variability is associated with the variety of coordination patterns used to complete tasks^65,66^. For the current study, we calculated RMSEs using a linear regression model and considered the parameter as an indicator of MV. The reason that we considered RMSE as a measure of MV was that the Euler angle values measure movement acceleration and deceleration. Thus, the RMSE represents the difference between the generated linear regression model (fitted curve) and the obtained Euler angle data, which indicate the ideal movement trajectory and the raw data associated with shoulder movement, respectively. In the current study, MV during the abduction and adduction phases might indicate movement smoothness, while MV during the holding phase might indicate movement endurance.

#### Movement time (MT)

MT refers to the time required to execute a movement and is defined as the interval between movement onset and movement offset, representing temporal efficiency^107–109^. In the present study, MT included the duration of shoulder abduction (time between the onset of arm raising and attaining the maximum shoulder angle, SP to FP1), shoulder holding (time of maintaining the maximum shoulder angle, FP1 to FP2), and shoulder adduction (time between the onset of arm lowering and back to the initial position, FP2 to EP; see Fig. 8 for details). In general, MT is associated with muscle endurance, which is the ability of a muscle to sustain repetitive isometric or isotonic contractions^110^.

### Statistical analysis

The kinematic parameters from the affected side were used and categorised into the three phases of shoulder movement (abduction, holding, and adduction). A one-way repeated measures analysis of variance was performed to compare the kinematic parameters across the three cueing conditions (NAC, RAC, and MAC). All analyses were conducted using R (R Project for Statistical Computing, Vienna, Austria).

## Data Availability

The datasets generated and/or analysed during the current study are available from the corresponding author upon reasonable request.
